# Mitigating the Effects of Xuebijing Injection on Hematopoietic Cell Injury Induced by Total Body Irradiation with γ rays by Decreasing Reactive Oxygen Species Levels

**DOI:** 10.3390/ijms150610541

**Published:** 2014-06-12

**Authors:** Deguan Li, Lu Lu, Junling Zhang, Xiaochun Wang, Yonghua Xing, Hongying Wu, Xiangdong Yang, Zhexin Shi, Mingfeng Zhao, Saijun Fan, Aimin Meng

**Affiliations:** 1 Tianjin Key Lab of Radiation Medicine and Molecular Nuclear Medicine, Institute of Radiation Medicine, Academy of Medical Science and Peking Union Medical College, Tianjin 300192, China; E-Mails: lideguan@irm-cams. ac.cn (D.L.); lulu@irm-cams.ac.cn (L.L.); zhangjunling@irm-cams.ac.cn (J.Z.); wangxiaochun@irm-cams.ac.cn (X.W.); xyhandy@163.com (Y.X.); why999@irm-cams.ac.cn (H.W.); fansaijun@irm-cams.ac.cn (S.F.); 2 Department of Hematology and Oncology, the First Teaching Hospital of Tianjin University of Traditional Chinese Medicine, Tianjin 300193, China; E-Mails: yxdtcm@sohu.com (X.Y.); shzhx0604@163.com (Z.S.); 3 Department of Hematology and Oncology, Tianjin First Central Hospital, Tianjin 300192, China; E-Mail: zmfzmf@hotmail.com

**Keywords:** Xuebijing injection, hematopoiesis, ionizing radiation (IR), reactive oxygen species (ROS)

## Abstract

Hematopoietic injury is the most common side effect of radiotherapy. However, the methods available for the mitigating of radiation injury remain limited. Xuebijing injection (XBJ) is a traditional Chinese medicine used to treat sepsis in the clinic. In this study, we investigated the effects of XBJ on the survival rate in mice with hematopoietic injury induced by γ ray ionizing radiation (IR). Mice were intraperitoneally injected with XBJ daily for seven days after total body irradiation (TBI). Our results showed that XBJ (0.4 mL/kg) significantly increased 30-day survival rates in mice exposed to 7.5 Gy TBI. This effect may be attributable to improved preservation of white blood cells (WBCs) and hematopoietic cells, given that bone marrow (BM) cells from XBJ-treated mice produced more granulocyte-macrophage colony forming units (CFU-GM) than that in the 2 Gy/TBI group. XBJ also decreased the levels of reactive oxygen species (ROS) by increasing glutathione (GSH) and superoxide dismutase (SOD) levels in serum and attenuated the increased BM cell apoptosis caused by 2 Gy/TBI. In conclusion, these findings suggest that XBJ enhances the survival rate of irradiated mice and attenuates the effects of radiation on hematopoietic injury by decreasing ROS production in BM cells, indicating that XBJ may be a promising therapeutic candidate for reducing hematopoietic radiation injury.

## 1. Introduction

Total body irradiation (TBI) results in the dysfunction of multiple organs, including the hematopoietic, gastrointestinal and cerebrovascular systems [1,2]. Extensive studies have focused on the potency of various compounds against radiation injury [3,4,5]. Recently, natural compounds or extracts, including *Menthe arvensis* and *Panax ginseng*, have attracted increased attention for use in radiation protection and therapy because of their low toxicity [6,7,8]. Therefore, the screening of Chinese traditional medicine compounds may be a valid approach to discover radioprotective agents.

Xuebijing injection (XBJ) is a traditional Chinese medicine that has been approved for many years by the State Food and Drug Administration (SFDA) of China to clinically treat sepsis [9]. *Carthami flos*, *Paeoniae* Radix Rubra, Chuanxiong Rhizoma, *Salviae miltiorrhizae* and *Angelicae* *sinensis* Radix are the main constituents of XBJ. XBJ has been widely used in the clinic to treat sepsis and multiple organ dysfunctions [10,11,12]. Recent studies showed that XBJ reduced patient mortality due to sepsis by inhibiting T-cell growth [13]. In addition, active constituents of XBJ, such as paconiflorin, Danshensu and Hydroxysaffior Yellow A, inhibited p38 MAPK expression [14].

Hematopoietic injury is the most common side effects of TBI. Our prior studies have shown that MAPK inhibition mitigated hematopoietic radiation injury in irradiated mice [15,16]. However, the potential of XBJ to protect against radiation-induced hematopoietic injury remains unknown. Therefore, in this study, we investigated the potential use of XBJ to treat hematopoietic ionizing radiation (IR) injury by examining the effects of XBJ on the survival and hematopoiesis of mice exposed to IR.

## 2. Results and Discussion

### 2.1. XBJ Increased the 30-Day Survival Rate of Mice Exposed to a Lethal Dose of TBI

**Figure 1:**
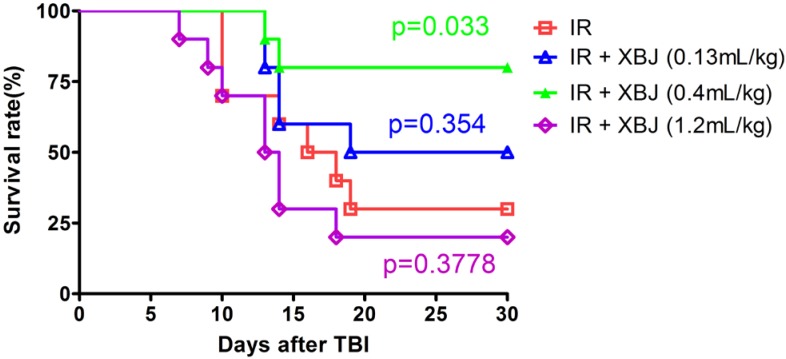
The survival of mice exposed to total body irradiation (TBI) (7.5 Gy) was increased by Xuebijing injection (XBJ) administration. After the mice had been exposed to 7.5 Gy TBI, the mice were treated by intraperitoneal (i.p.) injection of the vehicle as an irradiation control (IR, *n* = 10). XBJ was administered as described in the Experimental Section. The data are expressed as the percentage of surviving mice. The *p*-value indicates the significance of the difference between the mice treated with XBJ and the IR alone group and was determined by a log-rank test.

In the current study, the effect of XBJ on TBI-induced injury of ICR mice was first evaluated by determining the 30-day survival rate of mice after exposure to a lethal dose (7.5 Gy) of TBI. As shown in Figure 1, 7.5 Gy TBI resulted in 70% mortality in the IR group. The 30-day survival rates after TBI were 50%, 80% and 20% for the mice treated with 0.13, 0.4 and 1.2 mL/kg of XBJ, respectively. Compared with the control group, the survival rate of the mice after treatment with IR decreased significantly (*p* = 0.0011). The survival rate of mice treated with IR + XBJ (0.4 mL/kg) was higher than that of the IR group (*p* = 0.033), which demonstrates that 0.4 mL/kg XBJ had protected against IR-induced death in ICR mice. The 0.4 mL/kg dose of XBJ was used in subsequent experiments to treat mice with hematopoietic IR injury.

### 2.2. XBJ Mitigated IR-Induced Hematopoietic Cell Number Decreases

Exposure to IR induces a decrease in peripheral blood count and damage to the bone marrow. The recovery of hematopoiesis is an important factor in the treatment of radiation injury [17,18,19]. In this study, the effects of XBJ on bone marrow mononucleated cells (BMMNCs) were evaluated *in vitro*. After BMMNCs were treated with IR and XBJ, the viability of the cell was measured. The results showed that the viability of BMMNCs at 6 h was decreased by treatment with 1 and 4 Gy IR (Table 1). The viability of BMMNCs was increased by treatment with 1 and 5 μL/mL of XBJ. However, the viability of these cells was decreased after the cells were treated with 50, 100 and 200 μL/mL of XBJ. Similar results were obtained at 18 h (Table S1).

Hematopoietic parameters were examined nine days after 2 Gy TBI. As shown in Table 2, white blood cells (WBCs), RBCs, red blood cells (RBCs), hemoglobin (HGB), red blood cell specific volume (HCT) and platelets (PLT) in the 2 Gy group were significantly lower than in the control group. After 2 Gy TBI, WBCs, RBCs, HGB and HCT were increased in XBJ treated mice but remained lower than those of the controls.

**Table 1:** The viability (relative light unit × 10^3^) of bone marrow mononucleated cells (BMMNCs) 6 h after exposure to radiation.

XBJ (μL/mL)	0 Gy	1 Gy	4 Gy
0	130 ± 10	100 ± 3 ^#^	74 ± 3 ^#^
1	139 ± 9 *	107 ± 2 **^,#^	92 ± 7 **^,#^
5	143 ± 10 **	111 ± 2 *^,#^	89 ± 4 **^,##^
10	127 ± 7	109 ± 6 ^##^	91 ± 4 **^,##^
25	131 ± 8	99 ± 3 ^#^	89 ± 6 *^,##^
50	121 ± 8 *	93 ± 6 ^##^	77 ± 4 *^,##^
100	81 ± 5 **	73 ± 2 **^,#^	60 ± 3 *^,##^
200	26 ± 2 **	23 ± 2 **^,##^	19 ± 1 **^,##^

Bone marrow mononucleated cells were exposed to 0, 1 and 4 Gy of radiation after treatment with XBJ for 0.5 h. The viability of the cells was determined at 6 h after radiation exposure. The data are expressed as the means ± SEM (*n* = 4 for each group). * *p <* 0.05, ** *p <* 0.01 *vs*. control group; ^#^ *p* < 0.05, ^##^ *p <* 0.01 *vs*. the IR alone group.

**Table 2:** Peripheral blood cells were counted ninedays after 2 Gy TBI. WBC, white blood cell; RBC, red blood cell; HGB, hemoglobin; HCT, red blood cell specific volume; PLT, platelet.

Mice	WBCs (10^9^/L)	RBCs (10^12^/L)	HGB (g/L)	HCT (%)	PLT (10^9^/L)
Ctr	8.6 ± 1.5	9.0 ± 0.3	144.2 ± 2.8	35.0 ± 1.0	494.8 ± 51.1
0.4 mL/kg XBJ	9.8 ± 0.7	8.7 ± 0.3	138.2 ± 6.8	33.4 ± 1.8	522.4 ± 117.5
2 Gy	3.8 ± 0.4 **	7.6 ± 0.3 **	125.0 ± 6.5 **	30.2 ± 1.6 **	306.8 ± 41.8 **
2 Gy + 0.4 mL/kg XBJ	6.5 ± 1.6 *^,#^	8.2 ± 0.3 **^,#^	135.2 ± 6.6 *^,#^	32.6 ± 1.4 *^,#^	349.0 ± 88.7 *

Following exposure to 2 Gy TBI, the mice were treated by i.p. injection. A group of sham-irradiated control mice was included as a control (Ctr). Blood was collected and cells were counted after the mice were euthanized nine days after receiving 2 Gy TBI. The data are expressed as the means ± SEM (*n* = 5 for each group). * *p <* 0.05, ** *p <* 0.01 *vs*. the control group; ^#^ *p* < 0.05 *vs*. the IR group.

**Figure 2:**
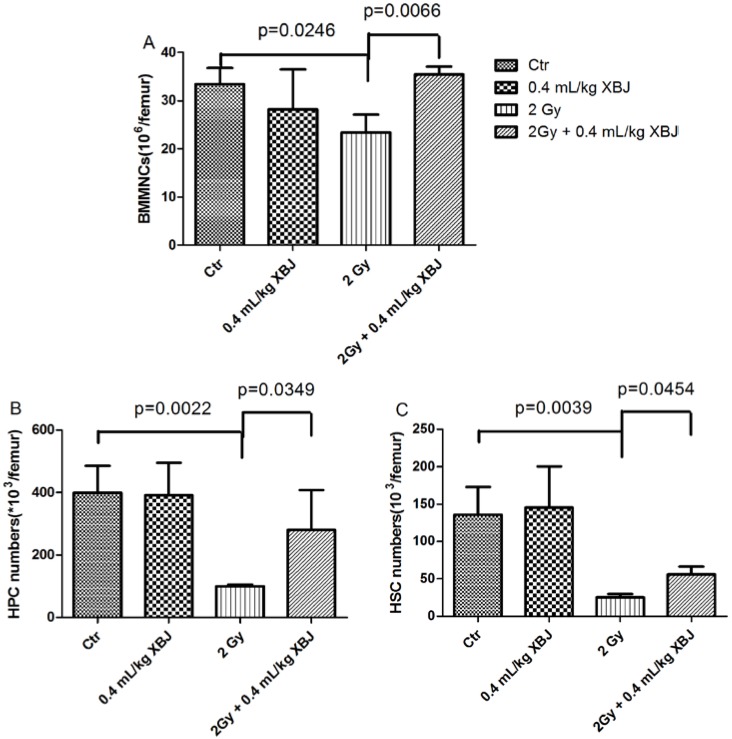
The effects of XBJ on the number of BMMNCs, hematopoietic progenitor cells (HPCs) and hematopoietic stem cells (HSCs) in irradiated mice. Mice were treated with i.p. injection of the vehicle or XBJ after exposure to 2Gy TBI, as described in the experimental section. A group of sham-irradiated mice was included as a control (Ctr) and received XBJ. BMMNCs (**A**) were counted after the mice were euthanized nine days post-radiation. The number of HPCs (**B**) and HSCs (**C**) was calculated using the formula: BMMNCs per femur × the HPC or HSC ratios detected by flow cytometry. The data are expressed as the means ± SD.

In addition, the number of BMMNCs, hematopoietic progenitor cells (HPCs) and hematopoietic stem cells (HSCs) from each femur was also determined (Figure 2). Compared with the controls (33.5 ± 3.3 × 10^6^ BMMNCs, 398.8 ± 86.9 × 10^3^ HPCs and 136.1 ± 36.7 × 10^3^ HSCs per femur), the number of BMMNCs (23.4 ± 3.7 × 10^6^ cells per femur), HPCs (100.4 ± 5.1 × 10^3^ cells per femur) and HSCs (25.5 ± 4.5 × 10^3^ cells per femur) was decreased in mice after receiving 2 Gy TBI (*p* < 0.05). The number of BMMNCs (35.5 ± 1.6 × 10^6^ cells per femur), HPCs (280.4 ± 126.7 × 10^3^ cells per femur) and HSCs (56.0 ± 10.5 × 10^3^ cells per femur) was higher in irradiated mice treated with XBJ than in vehicle-treated mice after TBI with 2 Gy (*p* < 0.05). These results showed that the treatment with XBJ promoted hematopoiesis after 2 Gy TBI.

### 2.3. XBJ Attenuated IR-Induced CFU-GM Reduction

**Figure 3:**
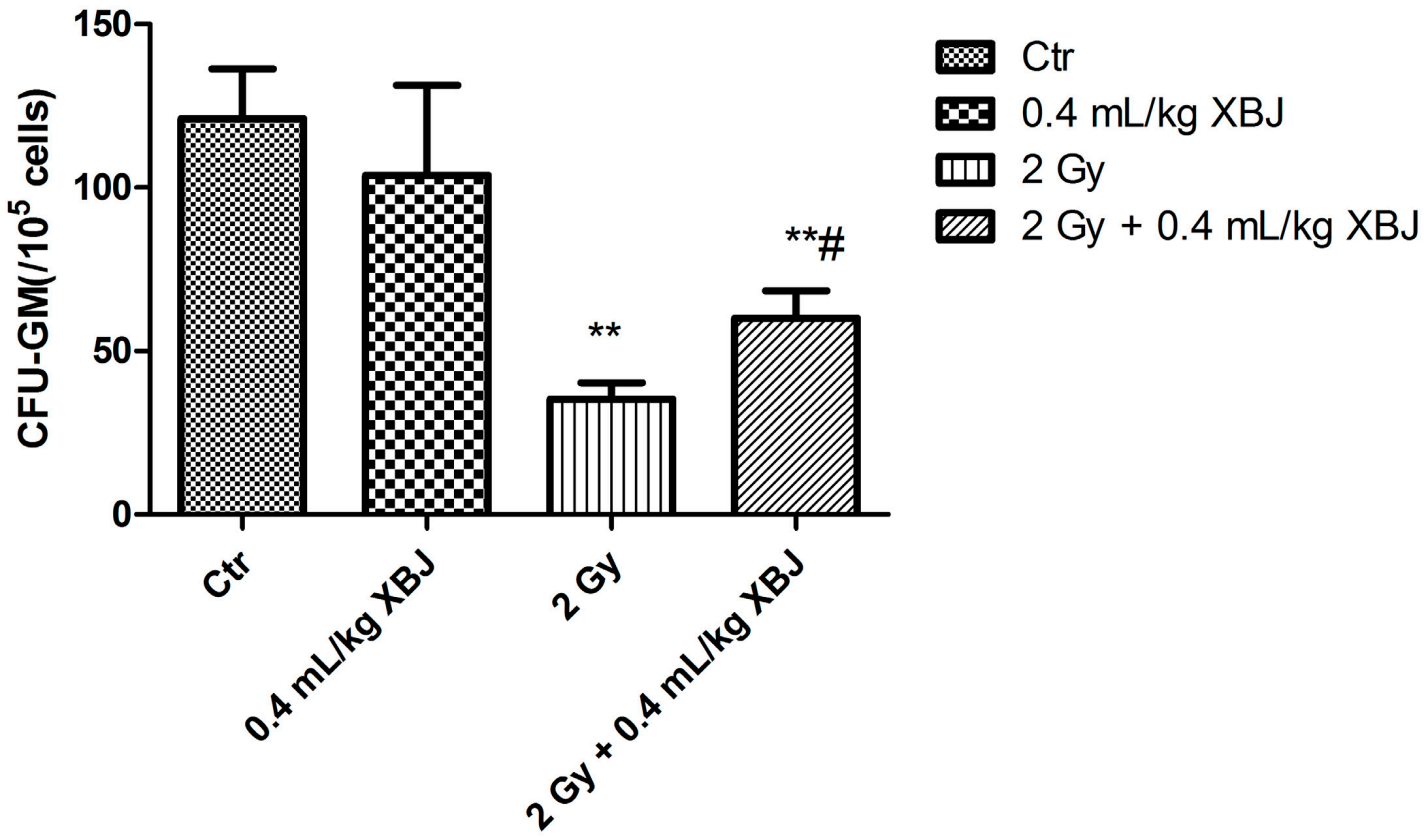
The ability of BMMNCs to form colonies, which was decreased by IR, was increased by XBJ administration. Mice were treated by i.p. injection of vehicle or XBJ after exposure to 2 Gy TBI. A group of sham-irradiated control mice was included as a control (Ctr) and XBJ. BMMNCs were collected from the mice nine days after 2 Gy TBI and cultured in MethoCult GF M3534 methylcellulose medium for CFU-GM analysis. The results are expressed as the number of CFU-GM per 10^5^ cells and are presented as the means ± SD. ** *p* < 0.01 *vs*. the sham-irradiated group and # *p* < 0.05 *vs*. the irradiated group.

Irradiation not only induces a reduction in the hematopoietic cell number, but also causes functional inhibition of cells. An assay to measure granulocyte-macrophage forming units (CFU-GM) was utilized to evaluate the function of hematopoietic cells. To determine whether XBJ increased hematopoiesis by promoting cellular function, we examined the effects of XBJ on the CFU-GM of BMMNCs. As shown in Figure 3, the amount of CFU-GM (35.3 ± 5.1 per 10^5^ cells) of BM cells from irradiated mice was significantly lower than that of non-irradiated mice (121.0 ± 15.3 per 10^5^ cells) (*p* < 0.01). The reduction in CFU-GM frequency was increased by treatment with 0.4 mL/kg XBJ (60.1 ± 8.3 per 10^5^ cells) (*p* < 0.05). This result suggests that the cellular functions affected by irradiation can be partially restored by XBJ treatment.

### 2.4. XBJ Attenuated IR-Induced BMMNC Apoptosis

**Figure 4:**
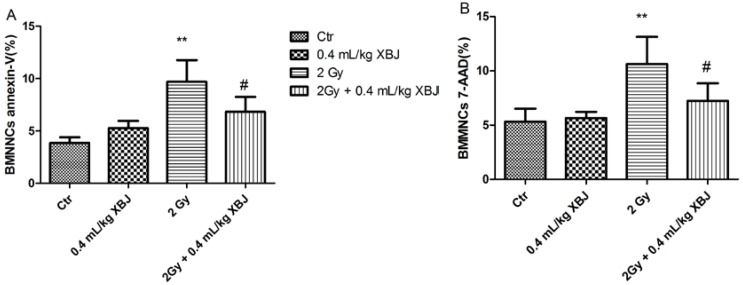
Apoptosis of BMMNCs induced by TBI was decreased by XBJ. (**A**) The effects of XBJ on Annexin-V-positive BMMNCs; (**B**) the effects of XBJ on 7-amino-actinomycin D (7-AAD)-positive BMMNCs. The data are expressed as the means ± SD. ** *p* < 0.01 *vs*. the control group; # *p* < 0.05 *vs*. the IR group.

To determine whether XBJ increased hematopoiesis by inhibiting apoptosis, BM cells were collected nine days after mice had been treated with 2 Gy TBI. Cells were stained with FITC-Annexin V and 7-amino-actinomycin D (7-AAD) and analyzed by flow cytometry (Figure S1). As shown in Figure 4A, the percentage of apoptotic BM cells (9.68% ± 2.07%) from irradiated mice was significantly higher than that of non-irradiated mice (3.85% ± 0.54%) (*p* < 0.01). This apoptosis was attenuated by treatment with XBJ (6.83% ± 1.41%, *p* < 0.05). Similar results were obtained in 7-AAD-positive cells (Figure 4B). These results showed that treatment with XBJ protected potential hematopoietic cells from radiation-induced injury by inhibiting both early and late apoptotic cell death.

### 2.5. XBJ Inhibited Radiation-Induced ROS Production

**Figure 5:**
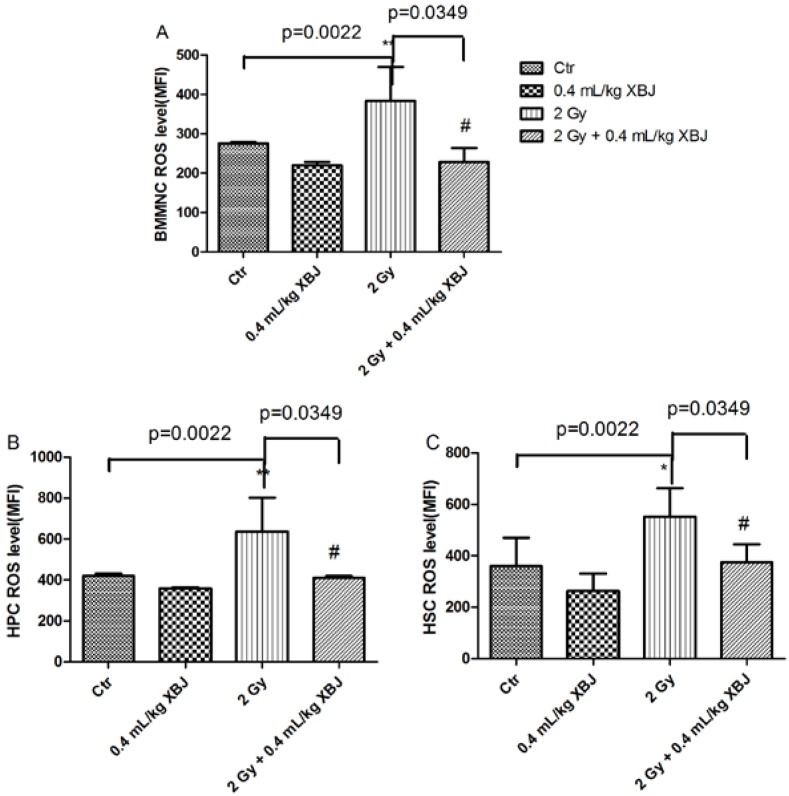
ROS levels were decreased by XBJ. (**A**) The ROS levels of BMMNCs; (**B**) the ROS levels of HPCs; (**C**) the ROS levels of HSCs. * *p* < 0.05, ** *p* < 0.01 *vs*. the control group; # *p* < 0.05 *vs*. the IR group.

We further determined the effects of XBJ on ROS production, which was detected by flow cytometry (Figure S2). For this *in vitro* assay, the change in ROS levels induced by IR in BMMNCs was not significant (Table S2). XBJ inhibited ROS production at doses of 5, 10 and 25 μL/mL in the non-irradiated groups. As shown in Figure 5, there was an increase in ROS levels in BMMNCs (383.3 ± 87.1), HPCs (636.5 ± 167.6) and HSCs (551.8 ± 112.0) in the IR group compared with the control group (275.3 ± 3.8, 419.8 ± 11.7 and 361.0 ± 108.8, respectively; *p* < 0.01). The ROS levels in BMMNCs (228.2 ± 35.6), HPCs (410.7 ± 10.1) and HSCs (375.0 ± 69.5) of the mice treated with XBJ were lower than those of IR mice (*p* < 0.05). These results suggest that XBJ inhibits BM apoptotic cell death by reducing ROS production.

**Figure 6:**
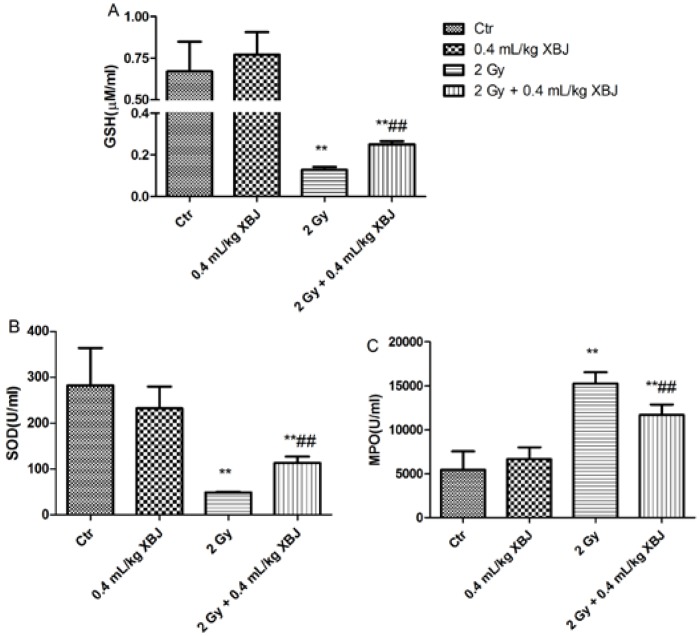
The effects of XBJ on the serum levels of GSH, SOD and MPO. Serum was collected from mice nine days after administration of 2Gy TBI, and the enzyme levels were measured with the appropriate kits. (**A**) GSH levels; (**B**) SOD levels; (**C**) myeloperoxidase (MPO) levels. The data were expressed as the means ± SD (*n* = 5). ** *p* < 0.01 *vs*. the control group; ## *p* < 0.05 *vs*. the IR group.

In this study, the serum levels of protective enzymes, such as SOD and GSH in serum, were also measured. As shown in Figure 6A,B, there was a reduction in GSH (0.13 ± 0.01 M) and SOD (48.9 ± 1.1 U/mL) in the serum of the IR group compared with that of the control group (0.67 ± 0.18 M and 282.7 ± 80.9 U/mL, respectively, *p* < 0.01). Compared with the IR group, treatment with XBJ elevated serum GSH (0.25 ± 0.01 M) and SOD (112.9 ± 14.1 U/mL, *p* < 0.01) levels. Myeloperoxidase (MPO) plays an important role in ROS production. After administering 2 Gy TBI, the serum levels of MPO (15.263 ± 1273 U/mL) were significantly elevated compared with those of the controls (Figure 6C, 5439 ± 2102 U/mL, *p* < 0.01). This elevation was attenuated by XBJ treatment (11.690 ± 1180 U/mL, *p* < 0.01). The results indicate that XBJ may inhibit ROS production by decreasing MPO release.

In this study, we first evaluated the overall effect of XBJ on the survival of mice exposed to IR [20,21]. We found that 0.4 mL/kg of XBJ elevated the 30-day survival rate of mice by up to 80%, which demonstrated that XBJ has a marked protective effect on IR-induced death in ICR mice. There was no protective effect on the survival rate observed for mice treated with 1.2 mL/kg XBJ, which was consistent with the results of *in vitro* cell viability assays. The results of these cell viability assays suggested that XBJ had protective effects at low concentrations. The cytotoxicity of XBJ at high concentrations might be due to its effects on the pH of the culture medium. XBJ may exert its protective effects on mice through its active components and through its metabolism by the body.

Based on these observations, we further examined peripheral blood. Parameters, such as WBCs and RBCs, increased significantly compared with the IR alone group when mice were treated with XBJ. Exposure to IR induces blood changes and bone marrow cell damage [22,23]. In the current study, the number and function of BMMNCs, HPCs and HSCs were also measured, as performed previously [24]. We found that XBJ increased the number and the colony-forming ability of bone marrow cells, which suggested that XBJ not only protected cells from IR injury, but also elevated cell function. IR is a potent inducer of apoptosis in a variety of cells, including hematopoietic cells. In this study, the percentage of apoptotic BMMNC was measured by flow cytometry, and we concluded that XBJ decreased the amount of the apoptosis induced by IR. 

Radiation induces the production of various forms of ROS, including superoxide radicals, hydrogen peroxide and the hydroxyl radicals, which contribute to IR injury. ROS react with multiple cellular components, such as DNA, RNA and proteins, resulting in apoptosis, senescence or necrosis [25]. ROS have been shown to play an important role in IR injury [26]. In this study, we found that IR elevated ROS production in BMMNCs, HPCs and HSCs. Compared with the IR group, the ROS levels of BMMNCs, HPCs and HSCs in the XBJ treated mice decreased significantly, which suggests that XBJ protects bone marrow cells from IR injury by eliminating ROS.

Cellular ROS can be eliminated through several detoxification mechanisms provided by endogenous antioxidant enzymes and antioxidants [27,28]. Depletion of intracellular glutathione (GSH) and superoxide dismutase (SOD) levels has been implicated as a main cause of radiation-induced damage, whereas alterations in GSH and SOD are responsible for the radioprotective effects. In this study, the levels of SOD and GSH in serum were increased in mice treated with XBJ. In addition, the serum levels of MPO were also decreased by XBJ. The results indicate that XBJ may decrease ROS levels through an induction of GSH or/and SOD and an inhibition of decreased MPO production.

Jiang found that six active ingredients of XBJ inhibited NF-Κb [14]. Liu reported that XBJ inhibited TLR4-NF-κB-IL-1β signal transduction in rats [29]. These results suggested that XBJ may protect mice from IR injury by inhibiting NF-κB, and these results need to be further confirmed.

## 3. Experimental

### 3.1. Animals and Reagents

Male, ICR mice were purchased from Vital River (Beijing, China) at six to eight weeks of age. The animals were quarantined and allowed to acclimatize for 1 week. They were maintained in a room at 22 ± 2 °C, with a relative humidity of 50% ± 10%. All animal experiments were conducted in accordance with a protocol approved by the Institutional Animal Care and Use Committee (IACUC) of the Institute of Radiation Medicine, Chinese Academy of Medical Sciences. (NO. 1204)

XBJ was purchased from Tianjin Chase Sun Pharmaceutical Co., Ltd. (Tianjin, China). Biotin-CD5, Ter119, CD11b, CD45R/B220, Gr-1, APC-c-kit, PE-Cy7-Sca-1, streptavidin-APC-Cy7 and a FITC-Annexin V apoptosis detection kit were purchased from Ebioscience (San Diego, CA, USA). Glutathione (GSH), superoxide dismutase (SOD) and myeloperoxidase (MPO) detection kit instructions were purchased from ImmunoWay (Newark, DE, USA).

### 3.2. Irradiation

Mice were placed in ventilated Plexiglas containers and received total body irradiation (TBI) using ^137^Cs γ rays (Cammacell-40, Atomic Energy , Mississauga, ON, Canada) at a dosage of 1.0 Gy/min. Non-irradiated mice were also placed in identical containers for the same period without irradiation. After irradiation, animals were returned to the animal facility for daily observation and treatment, as described below. The mice received 7.5 Gy TBI in the survival experiments, and the mice in the remaining experiments received 2.0 Gy TBI. After treatment with different concentrations of XBJ for 0.5 h, bone marrow mononucleated cells were exposed to 0, 1, 4 Gy for *in vitro* experiments.

### 3.3. Cell Viability Assays

Bone marrow cells were flushed from mouse femurs with PBS after mice had been sacrificed. Next, the cells were exposed to irradiation with 0, 1 or 4 Gy after treatment with 0, 1, 5, 10, 25, 50, 100 or 200 μL/mL XBJ for 0.5 h. Cell viability was monitored after 6 h using the luminescence-based CellTiter Glo system (Promega, Madison, WI, USA), according to the manufacturer’s recommended protocols.

### 3.4. XBJ Administration

XBJ was intraperitoneally injected for 9 days after TBI. Mice used for survival experiments were divided into five groups: Group I (control, Ctr) and Group II (irradiation, IR) received intraperitoneal injections of 0.2 mL/mice of isotonic saline solution for 8 days until the mice were sacrificed; Group III (0.13) received IR + XBJ at 0.13 mL/kg day; Group IV (0.4) received IR + XBJ at 0.4 mL/kg·day and Group V (1.2) received IR + XBJ at 1.2 mL/kg·day. Mice in the remaining experiments were divided into four groups: Group I (Ctr); Group II (XBJ) at 0.4 mL/kg/day; Group III (IR); and Group IV (IR + XBJ at 0.4 mL/kg/day).

### 3.5. Peripheral Blood Cell and BM Mononucleated Cells (BMMNCs) Counts

One hundred microliters of peripheral blood were obtained from the orbital sinus using a micro-pipette coated with the anticoagulant, K_3_EDTA, 9 days after irradiation. The number of blood cells was counted using a Celltac E hemocytometer (Nihon Kohden, Japan). The cell counts included white blood cells (WBCs), red blood cells (RBCs), hemoglobin (HGB), red blood cell-specific volume (HCT) and platelets (PLT). Bone marrow cells were flushed from the bones, as described previously, and BMMNCs were counted using the hematology analyzer and expressed as 10^6^ cells/femur.

### 3.6. Detection of HPCs and HSCs

Bone marrow (BM) cells were incubated with biotin-conjugated antibodies specific for murine CD5, Ter119, CD11b, CD45R/B220 and Gr-1 on ice for 30 min. After washing, the cells were stained with streptavidin-APC-Cy7, Sca1-PE-Cy7 and ckit-APC; HPCs (lin^−^c-kit^+^Sca-1^−^) and HSCs (lin^−^c-kit^+^Sca-1^+^) were analyzed. The HPC and HSC numbers were calculated using the equation: percentage × BMMNCs/femur.

### 3.7. Colony-Forming Assays

Colony-forming assays were conducted by culturing BM cells in MethoCult GF M3534 methylcellulose medium (StemCell Technologies, Vancouver, BC, Canada). The colonies of granulocyte macrophage cells (expressed in CFU-GM) with more than 30 cells were counted according to the instructions. The results are expressed as the numbers of CFU-GM (/10^5^ cells).

### 3.8. Intracellular ROS Analysis

After BM cells were stained with the lineage markers, Sca-1 and c-kit, as described above, the cells were incubated with 2,7-dichlorodihydrofluorescein diacetate (DCFDA ,10 µM) for 30 min at 37 °C. The levels of intracellular ROS in BMMNCs, HPCs and HSCs were analyzed by measuring the mean fluorescence intensity (MFI) of DCF using flow cytometry [30,31]. For each sample, a minimum of 1000 HSCs were acquired. For *in vitro* assays, the BMMNCs were incubated with DCFDA (10 µM) for 30 min at 37 °C after the cells were exposed to 0, 1 or 4 Gy for 6 h. The levels of intracellular ROS in BMMNCs were analyzed by measuring the MFI of DCF using flow cytometry.

### 3.9. Assay of SOD, GSH and MPO Levels in Serum

The remaining blood was collected in tubes and stored at room temperature for 4–6 h. The bloods were then centrifuged at 4000 rpm for 15 min, and the serum was collected and preserved at −80 °C. Levels of glutathione (GSH), superoxide dismutase (SOD) and myeloperoxidase (MPO) in the serum were detected according to the kit instructions (ImmunoWay, Newark, DE, USA).

### 3.10. Statistical Analysis

Data are presented as the means ± SD. The 30-day survival curves were compared using Kaplan–Meier methods with a log-rank test. Differences between groups were calculated using the Student’s *t*-test. A *p-*value of less than 0.05 were considered statistically significant.

## 4. Conclusions

In conclusion, XBJ mitigates the effects on hematopoiesis induced by TBI and may protect hematopoietic cells from IR injury by decreasing ROS levels. These data suggest that XBJ may be used to treat hematopoietic damage induced by ionizing radiation in the clinic.

## Supplementary Files

Supplementary File 1 Supplementary Information (PDF, 2078 KB)
