# Supplementary material for: Mitigating the Effects of Xuebijing Injection on Hematopoietic Cell Injury Induced by Total Body Irradiation with γ rays by Decreasing Reactive Oxygen Species Levels
Source: Int J Mol Sci. 2014 Jun 12;15(6):10541–53. doi: 10.3390/ijms150610541 (PMC4100167; doi:10.3390/ijms150610541)
Supplement: Supplementary File 1 — Supplementary Information (PDF, 2078 KB) [file ijms-15-10541-s001.pdf]

Supplementary Information

Table S1. The viability (RLU × 10^3) of BMMNCs 18 h after exposure to radiation.

XBJ (μL/mL)	0 Gy	1 Gy	4 Gy
0	77 ± 4	57 ± 1	41 ± 1
1	89 ± 5 **	61 ± 1 **,#	47 ± 3 *,#
5	88 ± 2 *	57 ± 2 ##	42 ± 2 ##
10	79 ± 2	50 ± 3 ##	38 ± 2 ##
25	64 ± 2 *	40 ± 2 **,#	33 ± 1 **,##
50	37 ± 2 **	26 ± 1 ##	23 ± 1 **,##
100	15 ± 1 **	14 ± 0 **,#	12 ± 0 **,##
200	4 ± 0 **	3 ± 0 **,##	3 ± 0 **,##

Bone marrow mononucleated cells were exposed to 0, 1 and 4 Gy of radiation after treatment with XBJ for 0.5 h. The viability of the cells was determined at 6 h after radiation exposure. The data are expressed as the means ± SEM ($n = 4$ for each group). * $p < 0.05$, ** $p < 0.01$ *vs*. control group; # $p < 0.05$, ## $p < 0.01$ *vs*. the IR alone group.

Table S2. The ROS levels of BMMNCs 6 h after exposure to radiation.

XBJ (μL/mL)	0 Gy	1 Gy	4 Gy
0	3429 ± 44	3494 ± 104	3564 ± 56
1	3584 ± 75	3488 ± 116	3249 ± 111 *
5	3232 ± 41 *	3842 ± 157 *,#	4291 ± 135 **,##
10	3142 ± 83 *	3330 ± 129	3518 ± 12 #
25	2711 ± 24 **	2927 ± 51 **,##	3346 ± 79 *,#

Bone marrow mononucleated cells were exposed to 0, 1 and 4 Gy irradiation after treated with XBJ for 0.5 h. Then, the ROS levels of cells were detected by flowcytometry at 6 h after irradiation exposure. The data were expressed as mean ± SEM (n = 3 for each group). * $p < 0.05$, ** $p < 0.01$ *vs*. the control group; # $p < 0.05$, ## $p < 0.01$ *vs*. the IR alone group.

Figure S1. Representative graph for apoptosis analysis. Mice were treated with i.p. injection of the vehicle or XBJ after exposure to 2 Gy TBI, as described in the Experimental Section. BMMNCs were collected from the mice after euthanization nine days after 2 Gy TBI and incubated with FITC-Annexin V and 7-AAD, as per the manufactures instructions. Then, the BMMNCs were detected by flowcytometry and analyzed by the requisite software. (**A**) Apoptosis of control cells; (**B**) apoptosis of |IR cells.

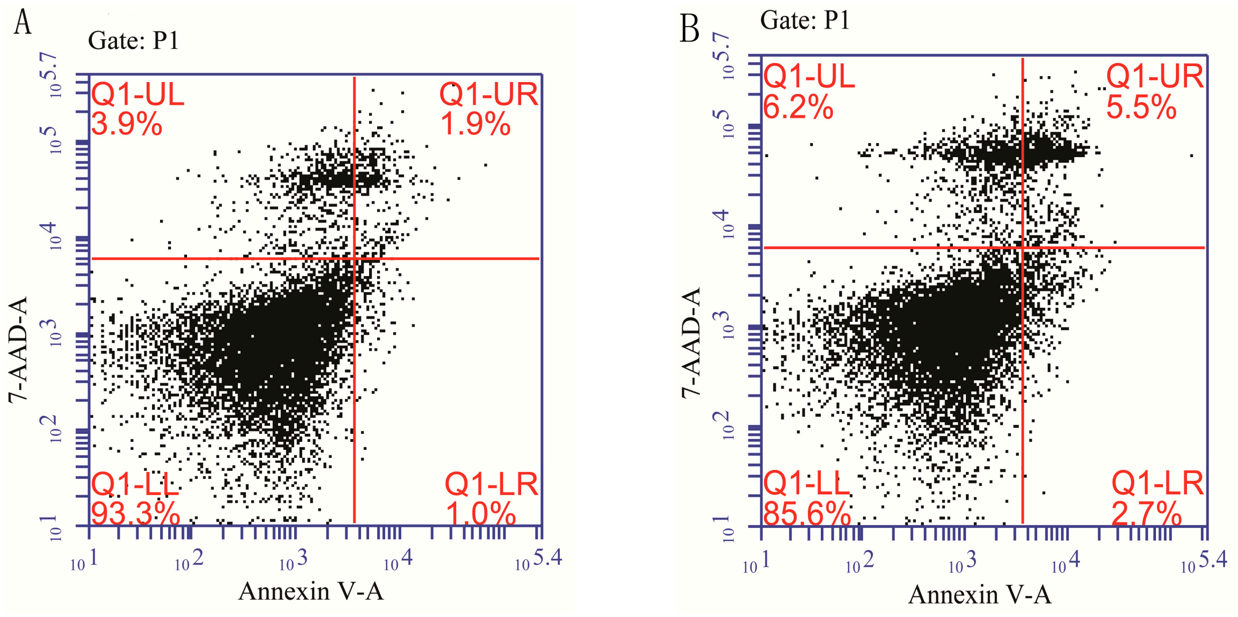

Figure S2. Representative ROS measurement. Mice were treated with i.p. injection of the vehicle or XBJ after exposure to 2 Gy TBI, as described in the Experimental Section. BMMNCs were collected from the mice after euthanization nine days after 2 Gy TBI and incubated antibodies, such as biotin-lineage (CD5, B220, Ter-119, CD11b, Gr-1), APC-c-kit, PE-cy7-Sca-1 and streptavidin-APC-cy7, as per the manufactures' instructions. Then, the cells were detected by flowcytometry and analyzed by the requisite software. (**A**) Bone marrow mononucelated cells (gated); (**B**) adhesion cells removement; (**C**) lineage negative cells gated; (**D**) HSC and HPC gated; (**E**) representative HSC ROS detection; (**F**) representative HPC ROS detection.

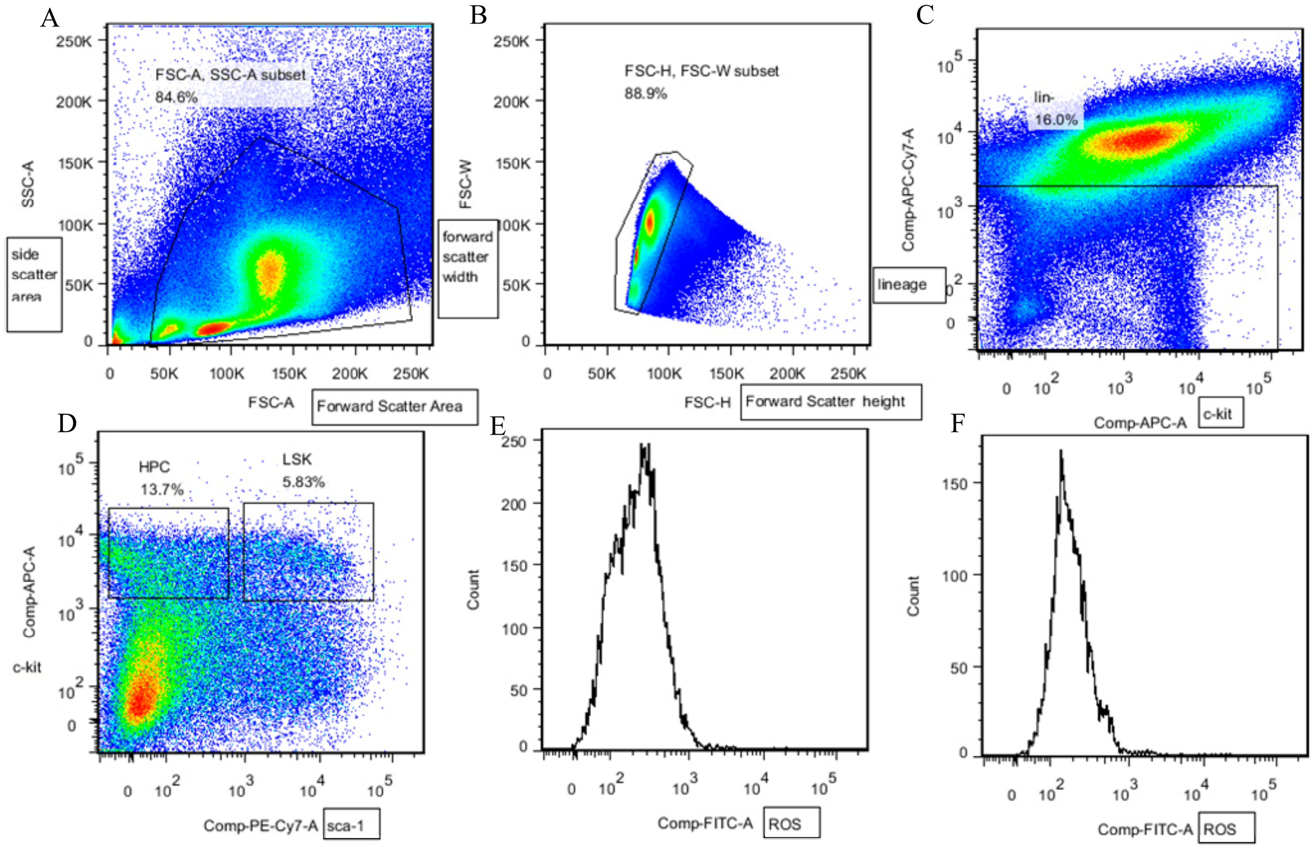

© 2014 by the authors; licensee MDPI, Basel, Switzerland. This article is an open access article distributed under the terms and conditions of the Creative Commons Attribution license (http://creativecommons.org/licenses/by/3.0/).
